# Neurovascular unit, neuroinflammation and neurodegeneration markers in brain disorders

**DOI:** 10.3389/fncel.2024.1491952

**Published:** 2024-10-25

**Authors:** Duraisamy Kempuraj, Kirk D. Dourvetakis, Jessica Cohen, Daniel Seth Valladares, Rhitik Samir Joshi, Sai Puneeth Kothuru, Tristin Anderson, Baskaran Chinnappan, Amanpreet K. Cheema, Nancy G. Klimas, Theoharis C. Theoharides

**Affiliations:** ^1^Dr. Kiran C. Patel College of Osteopathic Medicine, Institute for Neuro-Immune Medicine, Nova Southeastern University, Ft. Lauderdale, FL, United States; ^2^College of Psychology, Nova Southeastern University, Ft. Lauderdale, FL, United States; ^3^Miami VA Geriatric Research Education and Clinical Center (GRECC), Miami Veterans Affairs Healthcare System, Miami, FL, United States; ^4^Department of Immunology, Tufts, University School of Medicine, Boston, MA, United States

**Keywords:** blood-brain barrier disruption, glial cells, neuroinflammatory biomarkers, neurodegenerative disorders, neurofilament light, neurovascular unit, tight junction proteins

## Abstract

Neurovascular unit (NVU) inflammation via activation of glial cells and neuronal damage plays a critical role in neurodegenerative diseases. Though the exact mechanism of disease pathogenesis is not understood, certain biomarkers provide valuable insight into the disease pathogenesis, severity, progression and therapeutic efficacy. These markers can be used to assess pathophysiological status of brain cells including neurons, astrocytes, microglia, oligodendrocytes, specialized microvascular endothelial cells, pericytes, NVU, and blood-brain barrier (BBB) disruption. Damage or derangements in tight junction (TJ), adherens junction (AdJ), and gap junction (GJ) components of the BBB lead to increased permeability and neuroinflammation in various brain disorders including neurodegenerative disorders. Thus, neuroinflammatory markers can be evaluated in blood, cerebrospinal fluid (CSF), or brain tissues to determine neurological disease severity, progression, and therapeutic responsiveness. Chronic inflammation is common in age-related neurodegenerative disorders including Alzheimer’s disease (AD), Parkinson’s disease (PD), and dementia. Neurotrauma/traumatic brain injury (TBI) also leads to acute and chronic neuroinflammatory responses. The expression of some markers may also be altered many years or even decades before the onset of neurodegenerative disorders. In this review, we discuss markers of neuroinflammation, and neurodegeneration associated with acute and chronic brain disorders, especially those associated with neurovascular pathologies. These biomarkers can be evaluated in CSF, or brain tissues. Neurofilament light (NfL), ubiquitin C-terminal hydrolase-L1 (UCHL1), glial fibrillary acidic protein (GFAP), Ionized calcium-binding adaptor molecule 1 (Iba-1), transmembrane protein 119 (TMEM119), aquaporin, endothelin-1, and platelet-derived growth factor receptor beta (PDGFRβ) are some important neuroinflammatory markers. Recent BBB-on-a-chip modeling offers promising potential for providing an in-depth understanding of brain disorders and neurotherapeutics. Integration of these markers in clinical practice could potentially enhance early diagnosis, monitor disease progression, and improve therapeutic outcomes.

## Introduction

Neuroinflammatory and neurodegenerative disorders are characterized by the presence of acute and chronic neuroinflammatory responses in the brain. Neuroinflammatory response is the initial response to protect the brain against damage, infection such as microbial infections/sepsis or exposure to toxins by activated glial cells and neurons (Kempuraj et al., 2020a; Gao and Hernandes, 2021; Tran et al., 2022). However, excessive and persistent glial cell activation leads to chronic neuroinflammation-associated neurodegeneration and increases disease severity of neurodegenerative disorders (Le Thuc et al., 2015; Kempuraj et al., 2016). The neuroimmune system is implicated in the development, normal functioning, aging, and integrity of the central nervous system (CNS) (Hickman et al., 2018). Chronic disorders such as Alzheimer’s disease (AD), Parkinson’s disease (PD) and traumatic brain injury (TBI) are neuroinflammatory and neurodegenerative disorders with dysfunctional neurons, synapses, glial cells and their networks (Pathak et al., 2022). Conditions such as Gulf War Illness (GWI) and Myalgic encephalomyelitis/chronic fatigue syndrome (ME/CFS) are also chronic disorders that exhibit several neurological symptoms, neuroimmune dysfunction and neuroinflammation (Wirth et al., 2021; Cohen et al., 2024). The precise mechanisms underlying the pathogenesis of various neurodegenerative diseases are likely different and are currently not yet clearly understood. Different disease triggers can cause neuroinflammation and neuronal damage in different brain regions involving specific types of brain cells and pathways. Additionally, inflammatory mediators from peripheral inflammation can also influence neuroinflammation and neurodegeneration in the brain through a defective and vulnerable blood-brain barrier (BBB) (Kempuraj et al., 2017).

The BBB plays an important role in brain homeostasis by allowing selective molecules from peripheral blood into the brain parenchyma (Chin and Goh, 2018; Zapata-Acevedo et al., 2024). Neuroinflammation and neurodegenerative disorders disrupt the BBB, and increase permeability allowing the entry of immune cells, inflammatory mediators, toxic substances, and pathogens from the peripheral blood into the brain (Musafargani et al., 2020). Derangements and damage to the tight junction (TJ), adherens junction (AdJ), and gap junction (GJ) components of the BBB lead to increased BBB permeability, resulting in edema, increased neuroinflammation and neuronal damage in various brain disorders (Kempuraj et al., 2020a; Bhowmick et al., 2019). Neuroinflammation can lead to upregulation or downregulation of certain specific markers in different brain cells. Neuroinflammation can be beneficial by removing cellular debris and promoting the tissue repair process (Le Thuc et al., 2015). Neuroinflammation has also been shown to enable the proliferation and maturation of neuronal precursor cells, axonal regeneration, and remyelination over denuded axons (Yong et al., 2019). Damage/activation of glial cells, specialized brain endothelial cells, neurons, and BBB structure trigger the release of distinct markers from these cells into the cerebrospinal fluid (CSF) and blood that can be assayed by different procedures for the evaluation of disease status, progression and therapeutic efficacy. However, the dynamics of the BBB in various pathophysiological conditions are not yet clearly known. The development of BBB-on-a-chip modeling in the last decade has the potential for further understanding of BBB dynamics in pathophysiological conditions and neurotherapeutics (Peng et al., 2022; Ohbuchi et al., 2024). In this review, we present markers of neurons, glial cells, neurovascular unit (NVU), BBB proteins, neuroinflammation, and neurodegeneration associated with acute and chronic brain disorders.

## Neuroinflammation and neurodegeneration markers

**FIGURE 1:**
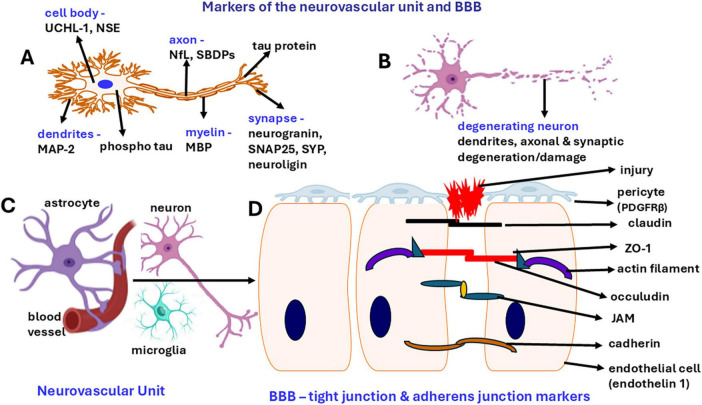
Schematic diagram shows neuronal markers in the neurovascular unit with BBB. BBB is a dynamic structure that functions as a gatekeeper. **(A)** Markers in neurons in different regions. **(B)** neurodegeneration of dendrites, axon, synaptic area. **(C)** NVU is comprised of micro blood vessels, endothelial cells, BBB, pericytes, neurons, astrocytes, and microglia. **(D)** BBB with endothelial cells, pericytes, tight junction and adherens junctions consisting of claudin, zonula occludens *(*ZO-1), occuludin, junctional adhesion molecule (JAM) and cadherin that regulates BBB permeability. The presence of increased levels of these markers in the blood or CFS indicates the damage to these structures. Derangement of BBB proteins can also cause BBB dysfunction.

Neurogenesis is a turnover process that generates new neurons during adulthood, maintaining the integrity of the brain. Neurodegeneration is a slow and progressive dysfunction, loss of axons and neurons, which is accelerated by the aging process as well as the neuroinflammatory process (Culig et al., 2022). Mature neuronal markers include nuclear protein neuronal nuclei (NeuN; nuclei), neuron-specific enolase (NSE; cell bodies/soma), neurofilament light (NfL; axons), TUJ1 (class III beta-tubulin; cytoskeleton), tau (axon, cell body, dendrites), spectrin breakdown products (SBDPs; axons), and microtubule-associated protein 2 (MAP2; dendrites) which indicate specific parts of the neuron or damage (Zetterberg and Blennow, 2016; Figure 1). Synaptic markers include synaptosomal-associated protein (SNAP25), synaptophysin (SYP), and neuroligin (Zetterberg and Blennow, 2016). Neurodegeneration can be assessed by neuronal markers MAP2, NfL, TUJ1, and SYP. However, certain markers such as amyloid precursor protein (APP), amyloid β (Aβ) and tau are more specific to AD pathology. Synaptic disorder, synaptic loss and cognitive decline are common manifestations of neurodegenerative disorders (Dejanovic et al., 2024). Neuronal damage, neurodegeneration and neuronal loss have been reported in AD, PD and TBI. Nearly 19.5% of soldiers deployed in Operation Iraqi Freedom (OIF) and Operation Enduring Freedom (OEF) were exposed to blast traumatic brain injury (bTBI) (Kempuraj et al., 2020a). Certain conditions such as TBI and stress are risk factors for the onset of progressive neurodegenerative disorders including AD and PD or dementia or can exacerbate the existing AD, PD pathologies and dementia (Kempuraj et al., 2020b; Brett et al., 2022). The levels of ubiquitin C-terminal hydrolase-L1 (UCH-L1) and glial fibrillary acidic protein (GFAP) in the blood are U.S. Food and Drug Administration (FDA)-approved biomarkers for mild TBI (mTBI) (Wang et al., 2021a). Certain brain injury/TBI markers include UCH-L1, NSE, erythrocyte membrane protein band 4.1 (EPB41) for cell body/soma injury, NfL, tau, myelin basic protein (MBP) for axonal injury, SNCA for synaptic injury, GFAP, S100B for glial cell injury and inflammatory cytokines and neurotoxic mediators (for inflammation) (Silvestro et al., 2024; Zetterberg and Blennow, 2016). Certain chronic neuroimmune conditions such as ME/CFS and GWI are associated with neuroinflammation but may not have apparent neurodegeneration (Cohen et al., 2024; O’Callaghan and Miller, 2019). Positron emission tomography (PET) and magnetic resonance spectroscopic (MRS) neuroimaging allow for a non-invasive “read” of the brain for neuroinflammatory processes and neuronal integrity in brain diseases (Van Der Naalt, 2015; Lee et al., 2024).

**FIGURE 2:**
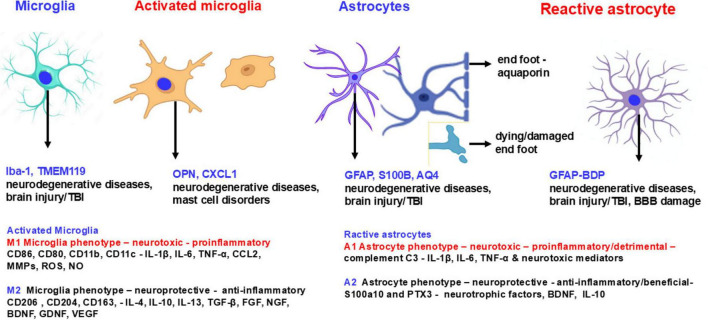
Schematic diagram shows microglial and astrocyte markers in normal, activated, anti-inflammatory and proinflammatory states. Microglia and astrocytes are activated in various neuroinflammatory and neurodegenerative disorders. Activated microglia and astrocytes express several markers that are also detected in biofluids. M1 microglia and A1 astrocytic phenotypes are neurotoxic and exacerbate neuroinflammation and neurodegeneration whereas M2 microglia and A2 astrocyte phenotypes are neuroprotective and protect the brain. These neurotoxic and neuroprotective glial cells express or secrete different molecules to exert detrimental or protective functions in the brain. AQ4, aquaporin 4; FGF, fibroblast growth factor; NO, nitric oxide; OPN, osteopontin; PTX3, pentraxin 3; ROS, reactive oxygen species; S100a10, S100 calcium-binding protein A10.

Activation of glial cells such as microglia and astrocytes lead to the release of molecules that trigger neuroinflammatory response and neuroinflammation. Both microglia and astrocytes can function either as neurotoxic (proinflammatory) M1 microglia and A1 astrocytes or as anti-inflammatory (neuroprotective) M2 microglia and A2 astrocytes phenotypes (Kwon and Koh, 2020; Guo et al., 2022). M1 microglia and A1 astrocytes release proinflammatory and neurotoxic molecules, whereas M2 microglia and A2 astrocytes produce neurotrophic and neuroprotective molecules that support neuronal growth and survival (Kwon and Koh, 2020). The M1/A1 or M2/A2 status (phenotype) of these cells can change during disease progression and can alter the severity of neuroinflammatory and neurodegenerative diseases (Kwon and Koh, 2020). Resting astrocytes (A0) become functional astrocytes (A1 and A2) by stimulation (Ding et al., 2021; Figure 2). Senescent dystrophic microglia have abnormal morphology with deramification (thin and short branches) and fragmented cytoplasm (Woollacott et al., 2020). The number of dystrophic microglia increases in neurodegenerative disorders such as AD in which many microglia are dysfunctional and senescent (Woollacott et al., 2020; Shahidehpour et al., 2021). Neuroinflammatory and neurodegenerative conditions impact the NVU which consists of microvascular specialized endothelial cells with BBB complex, pericytes and astrocytes (Bhowmick et al., 2019; Kempuraj et al., 2020a; Kempuraj et al., 2024). Disruption of NUV and BBB, glial activation and dementia have been reported in the recent coronavirus disease 2019 (COVID-19)/Long COVID conditions caused by infection with severe acute respiratory syndrome coronavirus 2 (SARS-CoV-2) (Kempuraj et al., 2024; Owens et al., 2024; Theoharides and Kempuraj, 2023; Shi et al., 2023; Zingaropoli et al., 2022). Inflammation in the brain activates glial cells to release inflammatory mediators which activate endothelial cells to express adhesion molecules and attract the peripheral blood leukocytes to the inflammatory site in the brain. Activated endothelial cells lead to loss of vascular integrity, increased adhesion molecule expression and cytokine and chemokine release including C-C motif ligand 2 (CCL2), CCL3, and interleukin-8 (IL-8) (Theofilis et al., 2021; Alsbrook et al., 2023). Cerebral endothelial cells express toll-like receptors (TLRs), chemokine receptors C-X-C motif chemokine receptor 1 (CXCR1), CXCR2, CXCR3, CCR3, CXCR4, and tumor necrosis factor receptors (TNFRs) TNFR1 and TNFR2. Pericytes cover the micro vessels in the brain and express various contractile and cytoskeleton proteins such as α-smooth muscle actin, nestin, myosin, vimentin, and desmin, cell surface neural/glial antigen 2 (NG2), platelet derived growth factor receptor beta (PDGFRβ), cluster of differentiation 13 (CD13), and CD146 (Alarcon-Martinez et al., 2021). Pericytes play a role in regulating the BBB, angiogenesis, removal of toxins, blood flow, stem cells, and neuroinflammation (Bhowmick et al., 2019). Pericytes can differentiate into microglia-like cells with phagocytic activity indicating that pericyte loss may increase leukocyte infiltration (Alsbrook et al., 2023). Additionally, pericytes can express TLR4 and exert a proinflammatory response. Pericyte damage can lead to BBB dysfunction allowing the influx of neurotoxic molecules in the brain from the peripheral blood. Astrocytes are the most abundant cells in the brain and are involved in the formation, maintenance and BBB permeability (Schiera et al., 2024; Rauf et al., 2022). Increased GFAP expression, an astrocyte marker, activates astrocytes and releases IL-1β, IL-6 and TNF (Giovannoni and Quintana, 2020). Astrocytes also induce anti-inflammatory effects and regulate neurotransmitter homeostasis such as glutamate. Peripheral inflammation may lead to brain endothelial activation, allowing peripheral blood inflammatory factors to enter the brain, activate perivascular macrophages and microglia, and initiate neuroinflammation without any primary injury or disease in the brain (Mayer and Fischer, 2024). Microglia are the primary innate/resident immune cells in the brain that first respond to injuries in the brain (Rauf et al., 2022). Microglia constantly sense changes in the brain tissue microenvironment for housekeeping function that helps neuronal health and functions (Mayer and Fischer, 2024). Microglia can express inflammatory cytokines and chemokines such as TNF, IL-1, IL-6, CCL2, and IL-18 to stimuli and they also express activation marker sTREM2 (soluble triggering receptor expressed on myeloid cells 2).

Understanding NVU/BBB dynamics in the brain’s pathophysiological conditions will improve the treatment options for brain disorders. In addition to the cells in the brain, infiltration of immunocytes, cytokines, chemokines and neurotoxic molecules from the periphery also activate glial cells, further releasing additional inflammatory mediators that accelerate neuroinflammation in the brain. Pathogenic substances that enter from the periphery to the brain also enhance inflammatory response in the brain (Kempuraj et al., 2017). There are several types of biomarkers including immunochemical analysis in tissues that involve tissue biopsy or post-mortem tissue, blood (minimally invasive)/CSF (invasive) based biomarkers, physical by physical examination such as cognitive test, urine, and brain imaging such as MRI (Chahine et al., 2014). Extracellular vehicles (EVs) released from brain cells can be detected in the blood and CSF and used as a marker for brain disorders (Gamez-Valero et al., 2019; Ollen-Bittle et al., 2022). Additionally, genomic and proteomic analysis provides molecular level biomarkers with next-generation sequencing and mass spectrometry procedures for neurological disorders (Chase Huizar et al., 2020). Abnormally activated glial cells can secrete disease-specific proteins that can be used as a novel biomarker (Kim et al., 2020). Recent progress in proteomic research has the potential for the development of novel

biomarkers for brain disorders (Kim et al., 2020). MicroRNAs (miRNAs) play an important role in inflammatory response in neuroinflammation (Su et al., 2016). Liquid biopsies such as exosomal miRNA are important biomarkers for many diseases including neurological diseases (Malhotra et al., 2023; Zhou et al., 2024).

Table 1 provides various markers of neurons, astrocytes, microglia, neuroinflammation, neurodegeneration, the NVU, and the BBB complex with their dysfunctions and associated neuropathology. This table also includes some membrane proteins, secreted proteins, signaling proteins and structural proteins associated with brain cells. Effective neurotherapeutic options should ideally target the BBB complex, address the damage and derangement of BBB proteins, and reduce BBB dysfunction.

**TABLE 1:** Neurovascular/BBB and neuroinflammatory markers.

No.	Markers	Cells expressed and location	Marker type	Diseases/pathologies associated	References
1	NeuN (neuronal nuclear protein)	Specific neuronal marker (mature)−nuclear and perinuclear cytoplasm	Protein-neuronal marker-neuronal differentiation marker, mature neuron marker	Neuro-oncology, cancer diagnosis, cognitive impairment, dementia	Duan et al., 2016; Gusel’nikova and Korzhevskiy, 2015; Yang et al., 2024
2	MAP-2 (microtubule-associated protein 2)	Neurons (mature)/dendrites	Protein–neuron marker, found in somatodendritic compartment of neurons	Neurodegeneration	Nguyen et al., 2022; Johnson and Jope, 1992; Geisert et al., 1990
3	NFL (neurofilament light chain)	Neurons−myelinated axons−(mature)	Neuronal cytoskeleton protein in myelinated axons maintains neuronal shape and size, the transmission of a nerve impulse along axons, biomarker for neurodegeneration	Cognition, neurodegenerative indicators, monitor disease progression, MS, neurodegenerative dementia, stroke, TBI, amyotrophic lateral sclerosis and PD. Chronic traumatic encephalopathy (CTE), COVID	Ramani et al., 2021; Khalil et al., 2018; Shahim et al., 2020; Shahim et al., 2024; Mullard, 2023; Elahi et al., 2020; Zingaropoli et al., 2022
4	MBP (myelin basic protein)	Neurons-myelin sheath/in white matter−produced by oligodendrocytes	Protein, a marker of brain tissue injury, cerebral damage, and demyelination	MS/demyelinating diseases	Kim and Kim, 2024; Wasik et al., 2020; Bohnert et al., 2021
5	UCHL1 (ubiquitin C-terminal hydrolase L1)	Neurons (mature)-enzyme, highly expressed in the brain	A brain-specific enzyme, for axonal/neuronal repair after injury, axonal transport, memory, protect neurons	TBI, PD, AD -diffuse injuries	Hossain et al., 2024; Mi and Graham, 2023; Malhotra et al., 2024; Buneeva and Medvedev, 2024
6	βIII-tubulin (Tuj-1)	Differentiating neurons, early stages of neurogenesis and axonal growth	Earliest neuronal differentiation marker in CNS and PNS, neuronal identity marker	Tumor	Duly et al., 2022; Hausrat et al., 2021
7	TH (tyrosine hydroxylase)	Neuronal marker/substantia nigra/midbrain	B -tubulin protein family, enzyme	PD	Nagatsu et al., 2019; Thangavel et al., 2024
8	NPY (neuropeptide Y)	Neurons (GABAergic), cerebral cortex, hippocampus, hypothalamus, brain stem, thalamus	Peptide (abundant in CNS)	Neurogenesis, stress, anxiety, endothelial dysfunctions, brain disorders, depression	Shende and Desai, 2020; Morales-Medina et al., 2010; Thorsell, 2010
9	Neuroligins 1-4	Neurons-dendrite-postsynaptic, cortical astrocytes	Synaptic cell adhesion molecule, neuronal damage marker	Cognitive impairments,	Stogsdill et al., 2017; Sudhof, 2008; Sindi et al., 2014
10	Neurexin	Neurons-axon-pre-synaptic	Synaptic cell adhesion molecule	Cognitive impairments, autism spectrum disorder (ASD)	Craig and Kang, 2007; Zhang et al., 2023; Cao and Tabuchi, 2017; Sindi et al., 2014; Reissner et al., 2013
11	NSE (neuron specific enolase)	Neurons-neurons and their axonal and dendritic processes, astrocytes. The cytoplasm of neurons/neuronal damage indicator/tumor & prognosis	Enzyme, neural maturation index	Neuronal damage marker, cognitive disorder, neurotrauma, spinal cord injury, neuroendocrine tumors	Babkina et al., 2024; Haque et al., 2016; Dichev et al., 2020; Liu et al., 2024
12	Amyloid beta (Aβ) peptide 1-42 (from amyloid precursor protein)/ APs (amyloid plaques)	Peptide−extracellular	Peptide, cognitive dysfunction marker, AD biomarker	AD, Parkinson’s disease dementia (PDD)	Varesi et al., 2022; Teunissen et al., 2022; Yadollahikhales and Rojas, 2023
13	NFTs (neurofibrillary tangles)	Neurons-intracellular/lesions	Intracellular hyperphosphorylated tau-containing NFTs	AD, hallmark of AD	Korczyn and Grinberg, 2024; Mary et al., 2024; Mehta and Mehta, 2023; Kempuraj et al., 2019
14	Tau, phosphorylated Tau, total Tau	Neurons (mature)/microtube protein-accumulate & form NFTs	Protein, AD biomarker	AD, TBI	Teunissen et al., 2022; Varesi et al., 2022; Alonso et al., 2024; Granholm and Hamlett, 2024; Munoz Pareja et al., 2024
15	Total α-synuclein phosphorylated α-synuclein	Dopaminergic neurons, cortical neurons, substantia nigra, endothelial cells	Neuronal presynaptic cytoplasmic protein, synaptic signaling, non-fibrillar α-synuclein is cytotoxic	Neurodegeneration, PD	Praschberger et al., 2023; Morris et al., 2024; Negi et al., 2024
16	Lewy body (fibrillar aggregates)	Intraneuronal protein (α-synuclein) in nigrostriatal neurons	Aging, a hallmark of PD, a marker of neuronal degeneration	PDD dementia with Lewy bodies, Dementia with Lewy bodies (DLB), neurodegenerative disorder	Wakabayashi et al., 2013; Agarwal et al., 2024
17	Parkin	Primarily in brain cells – cytosolic, neuritis, synaptic vesicles	Mitochondrial metabolism, neuroprotective protein	PD	Wakabayashi et al., 2013; Song and Krainc, 2024
18	ApoE (apolipoprotein E)/ApoE e4	Astrocytes, macrophages, adipocytes	Protein, a risk factor for AD/Lipid/cholesterol transporter in blood	AD, BBB disruption, cognitive decline	Zhou et al., 2023; Jackson et al., 2022
19	GFAP (glial fibrillary acidic protein)	Astrocytes	Protein, maintain shape and motility of astrocytic process, BBB integrity	Focal brain lesions, TBI, an early biomarker for PD	Bhowmick et al., 2019; Shahim et al., 2024; Lotankar et al., 2017; Hossain et al., 2024; Elahi et al., 2020; Munoz Pareja et al., 2024; Sharma et al., 2022
20	GFAP-BDP (GFAP breakdown products)	Astrocytes	Astrocyte cytoskeleton, gliolysis	TBI, intracranial injury, PD	Lotankar et al., 2017; Okonkwo et al., 2013; McMahon et al., 2015; Boutte et al., 2016
21	DJ-1 (protein deglycase)	All cells including brain cells (neurons, glial cells)	Protein, neuroprotective role	Anti-oxidative properties, neurodegeneration	Lind-Holm Mogensen et al., 2023; Repici and Giorgini, 2019; Antipova and Bandopadhyay, 2017
22	S100β	Mature astrocytes that ensheath blood vessels, neurons	Cytoplasmic/nuclear protein, trophic and toxic effects, neurite outgrowth, prolonged neurite survival	Acute brain damage, CNS & BBB damage marker, TBI, neuropsychiatric disorders, neurodegeneration	Rothermundt et al., 2003; Hossain et al., 2024; Munoz Pareja et al., 2024
23	S100A8/S100A9 (MRP8, MRP9- calprotectin)	Neutrophils and monocytes/macrophages	100 family, Trigger chemotaxis and phagocytic migration	Inflammatory diseases, rheumatoid arthritis, trauma, stress, cancer	Shabani et al., 2018; Xia et al., 2024; Shepherd et al., 2006
24	AQ4 (aquaporin 4)	Astrocyte end-feet/blood vessel	Water channel protein, the most abundant molecule in the brain at the astrocytic membrane at BBB, adhesion molecule, synaptic plasticity	Edema, BBB damage, dementia, TBI, neuroinflammation, neurodegenerative disorders	Bhowmick et al., 2019; Lapshina and Ekimova, 2024; Nagelhus and Ottersen, 2013; Kitchen et al., 2020; Yang et al., 2016; Papadopoulos and Verkman, 2007; Bhend et al., 2023; Cibelli et al., 2021; Ikeshima-Kataoka, 2016
25	NGF (nerve growth factor)	Growth factor for nerve, from neurons of cortex and hippocampus	Regulate neuroimmune response	AD, wound repair	Rocco et al., 2018; Sims et al., 2022; Ding et al., 2020; Bruno et al., 2023
26	BDNF (brain-derived neurotrophic factor)	Major growth factor, a growth factor for neurons/neurogenesis (proliferation, differentiation and survival), neurotrophic, regulate synaptic connections, synaptic transmission, synaptic plasticity, released from neurons and glia	Growth factor, biomarker for PD, reduced in PD. neuronal maintenance, neuronal survival, plasticity, and neurotransmitter regulation.	AD, PD, Psychiatric and neurodegenerative disorders	Elahi et al., 2020; Albini et al., 2023; Lima Giacobbo et al., 2019; Zuccato and Cattaneo, 2009
27	GDNF (glial cell-derived neurotrophic factor)	For neuronal survival, the striatum, acts on dopaminergic/motor neurons	Growth factor, neuroprotection	PD (treatment)–neurodegenerative disorders	Allen et al., 2013; Cintron-Colon et al., 2020; Ford et al., 2023; Fusco and Paldino, 2024
28	SP (substance P)	Neurons, immune cells	Peptide, promotes wound healing, pain modulation	Anxiety disorder, major depressive disorder (MDD), post-traumatic stress disorder (PTSD), inflammation, nociception, Pain sensitivity, psychiatric conditions	Safwat et al., 2023; Mashaghi et al., 2016; Humes et al., 2024; Liao et al., 2024; Taracanova et al., 2018
29	NT (neurotensin)	Endothelial cells, peptide in CNS and GI tract, pre-post synaptic vesicles	Peptide/neurotransmitter, activate microglia	Pain, inflammation, stress-related disorder	Kyriatzis et al., 2024; Iyer and Kunos, 2021; Theoharides et al., 2016
30	Ng (neurogranin)	Neuron, synaptic marker, marker of synaptic degeneration	Protein, synaptic plasticity, synaptic regeneration	Synaptic dysfunction, synaptic damage, AD, PD, depression, TBI, stroke	Xiang et al., 2020; Lista and Hampel, 2017; Hellwig et al., 2015; Hawksworth et al., 2022
31	SNAP-25 (synaptosomal-associated protein-25)	Neuron, synaptic marker, neurotransmission	Protein	Synaptic dysfunction, synaptic damage, psychiatric disorders, AD, schizophrenia, epilepsy, attention deficient hyperactivity disorder	Hawksworth et al., 2022; Kadkova et al., 2019; Noor and Zahid, 2017
32	NTF3/4 (neurotropin-3/4)	Nerve growth factor, neuroplasticity, NGF family, induce the survival, development, and function of neurons	Neurotrophins	Neurodevelopmental disorders, major depressive disorder	Wysokinski, 2016
33	Fibronectin	Pericytes, endothelial cells, astrocytes (in vasculature in CNS)	Soluble glycoprotein, ECM protein, neuroprotection, axonal regeneration, BBB/vascular injury marker; extracellular protein, activates microglia and invading macrophages in the brain, wound healing	CNS-vascular injury/stroke	George and Geller, 2018; Patten and Wang, 2021; Dai et al., 2024; Wei et al., 2023a; Chu et al., 2023
34	GMF (glia maturation factor)	Astrocytes	Proinflammatory brain protein, activate microglia and macrophages	Neuroinflammation, neurodegenerative diseases, TBI	Fan et al., 2018; Kempuraj et al., 2018; Ahmed et al., 2020; Selvakumar et al., 2020a; Thangavel et al., 2017; Selvakumar et al., 2020b
35	CXCL1 C-X-C motif chemokine ligand 1 (fractalkine; FKN)	Neuron, astrocytes	Chemokine, microglia activation	Brain injury, neuroinflammation	Michael et al., 2020; Huang et al., 2023; Chen et al., 2023
36	Progranulin	Motor neurons	Neurotrophic factor/growth factor, anti-inflammatory protein, neuronal survival, role in synapse	Neurodegenerative diseases - dementia, Amyotrophic lateral sclerosis (ALS), AD	Wang et al., 2021b; Nabizadeh et al., 2024
37	MMPs (matrix metalloproteinases) MMP-9	CNS−from neurons endothelial cells, astrocytes, microglia, oligodendrocytes	Enzymes, beneficial synaptic plasticity, learning, and memory. critical for tissue formation, neuronal network remodeling, and BBB integrity, detrimental diseases, inflammation, neuronal death	Pathologic role in CNS diseases, neurodegeneration, AD, brain neurodegenerative diseases	Vafadari et al., 2016; Aksnes et al., 2023; Rempe et al., 2016; Norden et al., 2016; Sharma et al., 2022
38	Iba1 (ionized calcium-binding adaptor molecule 1)	Microglia, macrophages	A marker of microglia/macrophages	Neuroinflammation, indicator of microglia activation	Zhang et al., 2021; Thangavel et al., 2012
39	TMEM119 (transmembrane protein 119; Iba-1 & CD68 + microglia)	Microglia, a marker of microglia subset−M1 (CD80) & M2 (CD163, CD209)−brain or blood-derived	Only brain resident microglia express TMEM119 (not blood-derived macrophages)	AD (not in MS), TBI, ALS	Ruan and Elyaman, 2022; Satoh et al., 2016; Togawa et al., 2024
40	TREM2 (triggering receptor expressed on myeloid cells 2)	Microglia	Microglial function, receptor for a multitude of ligands enhancing their phagocytic activity	Neuroinflammatory diseases, AD, tau-mediated pathology	Pocock et al., 2024; Shi et al., 2024; Matteoli, 2024; Jain et al., 2023
41	P2RY12 (purinergic receptor P2Y, G-protein coupled 12)	Microglia, oligodendrocytes– receptor, immune cells	Receptor	Microglial activation, neuroinflammation, AD	Gomez Morillas et al., 2021; Kenkhuis et al., 2022; Cattaneo, 2015
42	CD11b	Microglia	Integrin molecule, role in cell migration, adhesion, and transmigration, bind to endothelial cells	Stroke, TBI	Korf et al., 2022; Kumar et al., 2017
43	CD80 (M1 microglia)	Microglia M1 type, immune cells	Membrane protein	Inflammatory type	Yamaguchi et al., 2024
44	CD162/CD209 (M2 microglia)	Microglia M2 type, surface receptor	Adhesion molecule	Anti-inflammatory type, immune response	Satoh et al., 2016
45	CD40	Microglia	Immunoregulatory protein	Neurological diseases, AD	Benveniste et al., 2004; Ots et al., 2022; Togo et al., 2000
46	CD45	Microglia	Pro-phagocytic and protective role	AD	Rangaraju et al., 2018
47	CD68	Microglia, monocytes/macrophages	Protein	ALS, carcinoma	Swanson et al., 2023; Waller et al., 2019
48	OX-42	Microglia	Microglia marker	Brain disorders	Robinson et al., 2014; Elkabes et al., 1996
49	Endothelin-1	Endothelial cells, some types of neurons, epithelial cells of the choroid plexus, and endothelial cells of micro vessels	Neuropeptide, neurovascular unit	Post COVID syndrome/Long COVID, neuroinflammation, neurodegenerative diseases, AD, TBI, ME/CFS	Banecki and Dora, 2023; Hostenbach et al., 2016; D’Orleans-Juste et al., 2019; Haffke et al., 2022; Custodia et al., 2023
50	vWF (von-Willebrand Factor)	Endothelial cells, Endothelial injury,	Neurovascular unit, endothelial cell marker	COVID-19, neuroinflammation, neurotrauma/TBI, angiogenesis, dementia, AD	Bhowmick et al., 2019; Wolters et al., 2018
51	Ang-2 (angiopoietin-2)	Endothelial cells, extracellular protein	Growth factor, promote neovascularization, role in angiogenesis and inflammation, neurovascular unit	Increase vascular permeability, BBB leakage, neuronal damage, AD, ME/CFS, Long COVID	Van Hulle et al., 2024; Haffke et al., 2022; Scholz et al., 2015; Ju et al., 2014; Hegen et al., 2004
52	Endosialin (CD248)/tumor endothelial marker 1 (TEM1)	Endothelial cells, tumor cells, vessels covering pericytes, pericytes	Endothelial marker, stromal fibroblast marker, pericyte proliferation	Tumor growth, brain tumor	Kontsekova et al., 2016; MacFadyen et al., 2005; Tomkowicz et al., 2010
53	ESM-1 (endocan)	Endothelial cells	Neurovascular unit	Post-COVID-19 syndrome, ME/CFS	Haffke et al., 2022
54	ICAM-1 (CD54/intercellular adhesion molecule-1)	Endothelial cells, astrocytes, microglia	Neurovascular unit	Inflammation, neuroimmune response; BBB, AD, PD	Sharma et al., 2022; Zhang et al., 2024; Janelidze et al., 2018
55	VCAM-1 (CD106/vascular cell adhesion molecule-1)	Endothelial cells	Neurovascular unit	Inflammation, neuroimmune response; BBB, AD	Sharma et al., 2022; Janelidze et al., 2018
56	NRP1 (neuropilin 1)	Endothelial cells	Neuronal axon growth, receptor for VEGF, vascularization	Angiogenesis, COVID-19, cancer/metastasis, vascular permeability, stroke	Domingues and Fantin, 2021; Al-Thomali et al., 2022; Cantuti-Castelvetri et al., 2020; Lim et al., 2021
57	PDGFRβ (platelet-derived growth factor-beta)	Pericytes	Neurovascular unit	Neuroinflammation	Bhowmick et al., 2019; Sharma et al., 2022; Kempuraj et al., 2021
58	NG2 (neural-glial factor/antigen 2)	Pericytes, other cells, non-neuronal cells, during development, NG2 cells can differentiate into oligodendrocytes, astrocytes and neurons. polydendrocytes, oligodendrocytes progenitor cells	NG2 cells may differentiate into neurons even in developed brain, NG2 cells also differentiate into astrocytes	Neuroinflammation, neurogenesis potential, AD, PD, MS, cerebrovascular disease	Hu et al., 2023; Zhang et al., 2022; Rigo et al., 2024; Mira et al., 2021; Wang and He, 2009; Bhowmick et al., 2019
59	CD13	Pericytes, endothelial cells, monocytes	Cell adhesion, monocyte/leucocyte trafficking across endothelial cells at the site of injury	Neuroinflammation	Mina-Osorio et al., 2008
60	ZO-1 (zonula occludens-1)	BBB-endothelium, microvascular endothelial cells	Tight junction protein, Zonula occludens-1 binds to the actin cytoskeleton for BBB integrity & permeability	Neuroinflammation, edema, BBB disruption, psychotic disorders, AD, TBI	Bhowmick et al., 2019; Sharma et al., 2022; Alluri et al., 2024; Aydogan Avsar and Akkus, 2024; Rochfort and Cummins, 2015; Kempuraj et al., 2021; Asghari et al., 2024; Dithmer et al., 2024
61	JAM-A (junctional adhesion molecule-A)	BBB-endothelium	Tight junction protein	Neuroinflammation, edema, BBB disruption, AD, TBI	Bhowmick et al., 2019; Kempuraj et al., 2021; Yeung et al., 2008; Dithmer et al., 2024
62	Claudins/Claudin-5	BBB-endothelium, microvascular endothelium	Tight junction protein	Neuroinflammation, edema, BBB disruption, neurological diseases, AD, TBI	Bhowmick et al., 2019; Haruwaka et al., 2019; Hashimoto et al., 2023; Wakayama et al., 2022; Asghari et al., 2024; Dithmer et al., 2024; Tachibana et al., 2024; Ohbuchi et al., 2024
63	Occludin	BBB-endothelium	Tight junction protein	Neuroinflammation, edema, BBB disruption, AD, TBI	Bhowmick et al., 2019; Asghari et al., 2024; Dithmer et al., 2024; Li et al., 2018
64	N-cadherin/VE-cadherin (vascular Endothelial cadherin)	BBB-endothelium	Adherens junction protein-assembly of AJ and BBB architecture, endothelial cell contact, endothelial injury marker of preclinical AD, cell proliferation, apoptosis	Neuroinflammation, edema, BBB disruption, AD, cognitive impairment	Asghari et al., 2024; Rho et al., 2017; Tarawneh et al., 2022; Bei et al., 2023
65	Connexin-43	BBB-endothelium; neurons, astrocytes & microglia form gap junction	Gap junction protein,	Neuroinflammation, edema, BBB disruption, promote immune quiescence of the brain by astroglial connection 43	Bhowmick et al., 2019; Boulay et al., 2015; Cibelli et al., 2021
66	IL-33	Damaged cells, immune cells, damaged astrocytes, Th2 cells, mast cells, endothelial cells	Cytokine, IL-1 superfamily, inflammatory, alarmin signal, neuroprotective effects, recruitment of microglia/macrophage, dual role as pro and anti-inflammatory effects	Tissue damage, activation of microglia, astrocytes, macrophage, endothelial cells and mast cells, neuroinflammation, cognitive impairments, TBI	Erenler and Baydin, 2020; Fu et al., 2016; Jiao et al., 2020; Vainchtein et al., 2018; Wicher et al., 2017; Reverchon et al., 2020; Rao et al., 2022
67	ST2 (soluble ST2)	Blood	IL-33 receptor, inflammatory, IL-33/ST2 axis protective through Treg	Inflammatory, tissue damage, AD, TBI	Fu et al., 2016; Xiong et al., 2014; Xie et al., 2022; Tan et al., 2023; Cao et al., 2018
68	IL-36	Brain cells, microglia, immune cells-monocytes, immune cells	Cytokine, IL-1 superfamily, inflammatory response, can activate microglia	Inflammation	van de Veerdonk et al., 2018; Zhou and Todorovic, 2021; Bozoyan et al., 2015
69	IL-37	PBMCs, macrophages, various tissues	Immunosuppressive cytokine, IL-1 superfamily, anti-inflammatory, neurotherapeutic agent	Inflammatory diseases, improve neuroprotection, suppress inflammation/innate immunity, stroke, AD, ASD	Brunt et al., 2023; Zhang et al., 2019; Lonnemann et al., 2022; Li et al., 2022; Tsilioni et al., 2019
70	IL-38	IL-1 family member, brain	Cytokine, IL-1 superfamily, anti-inflammatory	Suppress neuroinflammation, ASD. Cardiovascular and autoimmune diseases, chronic inflammatory diseases	van de Veerdonk et al., 2018; Tsilioni et al., 2020; Zare Rafie et al., 2021; Xu and Huang, 2018
71	ACE-2 (angiotensin-converting enzyme 2)	Receptor for SARS CoV-2, cell surface, endothelium, glial cells, neurons	Enzyme (protective)	COVID-19, long COVID, lung injury, renal dysfunction, protective role in fibrosis	Ahmad et al., 2022; Gupta et al., 2024; Tyagi et al., 2023; Varillas-Delgado et al., 2023; Tziolos et al., 2023; Wei et al., 2023b; Keller et al., 2023
72	VEGF (vascular endothelial growth factor)	Many cells-macrophages, mast cells	Vascular health, angiogenic factor, vasculogenesis, neuroprotective, rescue synaptic dysfunction, blood vessel formation, migration, proliferation of endothelial cells	Angiogenesis, cancer, arthritis, neuroinflammation, MS, AD	Sharma et al., 2022; Amini Harandi et al., 2022; Requena-Ocana et al., 2022; Elahi et al., 2020; Echeverria et al., 2017; Martin et al., 2021
	VEGF-A		Help recover the brain after severe injury, biomarker for cognitive impairment in alcohol use disorder	mTBI, cognitive function	Sun et al., 2024; Sun et al., 2022
	VEGFR2		Receptor for VEGF	AD	Cho et al., 2017; Harris et al., 2018
73	Osteopontin (OPN; CD44)	Microglia, mast cells, macrophages, activated T-cells, NK cells, and dendritic cells, in bone, astrocytes	Soluble cytokine, glycoprotein, adhesive protein in ECM, microglia activation marker, mast cell mediator, matrikine/soluble cytokine, regulate proliferation, migration and survival of astrocytes, regulate immune cell migration, communication, and response to brain injury	Mast cell disorders, injury, neuroinflammatory and neurodegenerative disorders, AD, ALS	Lin et al., 2023; George and Geller, 2018; Rosmus et al., 2022; Vay et al., 2021; Rentsendorj et al., 2018
74	Calprotectin ( S100A8/S100A9 (MRP8, MRP9)	Neutrophils and monocytes/macrophages	Protein, S100 family, leukocyte recruitment	Inflammatory diseases, trauma, stress, lung disorders, asthma, TBI	Shepherd et al., 2006; Yui et al., 2003; Kassianidis et al., 2022; Yang et al., 2021; Gruel et al., 2024
75	VIP (vasoactive intestinal polypeptide)	Neurons, endocrine and immune cells, cells in the intestine, pituitary	Hormone, neurotransmitter, neuromodulator, anti-inflammatory, regulate astrocytes and microglia, neuroprotective, anti-apoptotic, antioxidant, reduce Aβ plaques in AD	Osteoarthritis, neurodegenerative disorders, AD, PD, neuroinflammation	Korkmaz et al., 2019; Korkmaz and Tuncel, 2018; Carniglia et al., 2017; Morell et al., 2012; Mosley et al., 2019

The BBB is a crucial component of the NVU and plays an important role in the homeostasis of the brain. The NVU regulates BBB permeability, removal of toxic byproducts, and performs immune monitoring. BBB disruption and increased permeability are commonly observed in neurodegenerative disorders and neurotrauma, which increases BBB permeability causing or upregulating neuroinflammatory responses, neuroinflammation and neuronal loss (Yu et al., 2020). Therefore, we have highlighted recent advances in the study of BBB pathogenesis using the BBB-on-a-Chip model for CNS disorders and neurotherapeutics as briefly provided below.

## BBB-on-a-Chip for CNS disorders and neurotherapeutics

The integrity of the BBB is maintained by astrocytes, pericytes, endothelial cells, and neurons, TJ, AdJ and GP proteins of the BBB. This integrity is crucial for normal brain function. However, chronic damage to NVU and BBB components leads to BBB dysfunction, increased BBB permeability/leakage, and neuroinflammation in many neurodegenerative diseases (Ohbuchi et al., 2024; Yoon et al., 2021). Therefore, the ability to model BBB behavior and pathogenesis is essential for the understanding of CNS disorders and neurotherapeutics. Vascularization in the brain organoids can be induced by modeling BBB micro environment using chip technology (Urrestizala-Arenaza et al., 2024). The BBB-on-a-chip (BBB chip) micro-engineered laboratory technology is a powerful *in vitro* model closely resembling human BBB structure to study normal and diseased states (Peng et al., 2022; Berjaoui et al., 2024). BBB-on-a-chip technology has significantly improved over the last decade and has been used to study various neurological diseases including AD, PD and Multiple Sclerosis (MS) (Berjaoui et al., 2024; Kawakita et al., 2022; Yoon et al., 2021; Palma-Florez et al., 2023). Recently neuroinflammation on-a-chip for studying MS (Berjaoui et al., 2024) and neuropathogenesis-on-chips (Amartumur et al., 2024) technology have been reported. The recently developed *in vitro* microfluidic/microfluidic human BBB-on-a-chip modeling using brain endothelial cells, pericytes, and astrocytes tri-culture model along with immune cell (T-cell) migration will be highly useful for understanding BBB functions, permeability, the pathogenesis of brain diseases, and evaluation of neurotherapeutic drugs that target the BBB (Ohbuchi et al., 2024). However, a fully efficient BBB-on-a-Chip model is still not available to date. A recent article described the use of built-in sensors to characterize BBB models via quasi-direct current and electrical impedance measurements, and various biosensors for the detection of metabolites, drugs, or toxic agents (Kincses et al., 2023). Microfluidic BBB-on-a-Chip provides an engineered physiological microenvironment necessary for real-time monitoring of barrier properties using human cells (Musafargani et al., 2020). The availability of AXION Maestro Edge multiwell microelectrode array (MEA) system (Axion BioSystems, Atlanta, GA) coupled with NETRI’s NeuroFluidics devices (NETRI, Lyon, France) could significantly enhance brain-on-a-Chip and BBB-on-a-Chip modeling in the study of brain disorders including neurotrauma/TBI, and development of drugs that target the BBB (Cohen et al., 2024; Ohbuchi et al., 2024). In a 3D microfluidic system, brain organoids are placed at the center chamber and endothelial cells and pericytes are placed on the side channels to create a micro vascularization system (Urrestizala-Arenaza et al., 2024) In a study, BBBs-on-chips were exposed to TNF-α and IL-1β to mimic neuroinflammation and studies the BBBs-on-chip’s barrier function, cell morphology, increased expression of cell adhesion molecules, increased permeability, and T cell adhesion, extravasation, and migration across BBB-on-chips (Nair et al., 2023). Even though brain-on-a-chip technology advanced the understanding of BBB pathophysiology, these models are still in a preliminary state, and the neurospheroids are still far from the human brain tissue. Thus, new and more advanced clinically relevant bioengineered models of human brain-on-a-chip for drug efficacy evaluation are required (Staicu et al., 2021; Cui and Cho, 2022). We are currently working on a BBB-on-a-Chip model to create disease-surrogate models for different brain disorders. Further research advancement in the BBB-on-a-Chip model could enhance the understanding of BBB dynamics in both health and disease conditions and assist in the development of treatments that target the BBB.

## Conclusion

Neuroinflammation is a hallmark of many neurological disorders. Neuroinflammatory and neurodegenerative disorders are multifaceted processes involving the interaction of astrocytes, endothelial cells, neurons, microglia and infiltrating leukocytes as well as peripheral systems. Chronic release of neuroinflammatory mediators induces neuroinflammation, neurodegeneration, synaptic and neuronal loss and BBB dysfunction in the brain. Several molecules expressed by brain cells infiltrating peripheral leukocytes participate in the neuroinflammatory response in specific regions of the brain. Damage of NVU/BBB, TJ and AdJ proteins as well as neuroinflammatory markers could be assessed in the tissue as well as in CSF and blood though they are not specific to many brain disorders. Nevertheless, measuring such biomarkers is crucial for the diagnosis, severity assessment and treatment efficacy of various neurodegenerative disorders.
